# A Gem

**Published:** 1881-03

**Authors:** 


### A GEM.

The following, for pure knavery, is a gem.

"*Theoretically* it may do to assert that homœopathic physicians must never give but a single remedy in a case of disease, but *practically* it is quite a different matter"!

Evidently Dr. H. C. Wood had this gem before him when he wrote, "For a man to practice homœopathy at present necessitates that he be either ignorant, foolish or knavish—that is if it be knavish to live a lie."

